# Trends in Formulary Coverage of Nonprotected Class Drugs Granted FDA Accelerated Approval

**DOI:** 10.1001/jamanetworkopen.2025.36089

**Published:** 2025-10-07

**Authors:** Shelley A. Jazowski, Stacie B. Dusetzina

**Affiliations:** 1 Department of Social Sciences and Health Policy, Wake Forest University School of Medicine, Winston-Salem, North Carolina; 2 Department of Health Policy, Vanderbilt University School of Medicine, Nashville, Tennessee; 3 Vanderbilt-Ingram Cancer Center, Nashville, Tennessee

## Abstract

This cross-sectional study examines the trends in Medicare Part D formulary coverage of nonprotected class drugs granted accelerated approval by the US Food and Drug Administration.

## Introduction

The US Food and Drug Administration (FDA) Accelerated Approval Program has helped expedite the approval of treatments for serious health conditions.^1,2^ Most accelerated approval products are protected class drugs (eg, antineoplastic agents) with mandatory Medicare coverage^2,3^; however, plans’ formulary designs may limit coverage of and access to nonprotected class drugs granted accelerated approval.^3,4^ For example, tofersen received accelerated approval for amyotrophic lateral sclerosis in April 2023, yet Medicare Advantage plans classified it as experimental and investigational and automatically denied coverage.^5^ Medicare requires covered drugs to “be safe and effective and otherwise reasonable and necessary”^5^ and does not distinguish between drugs approved via the traditional or accelerated approval pathways^5^; therefore, our objective was to examine trends in Medicare Part D formulary coverage of nonprotected class drugs granted accelerated approval.

## Methods

We reviewed FDA reports to identify orally administered nonprotected class drugs granted accelerated approval from 2011 to 2024 (eMethods and eTable in Supplement 1). Next, we used quarterly Medicare Prescription Drug Plan Formulary files to examine initial and continued Medicare Part D coverage (from accelerated approval to quarter 1 of 2025). Given that nonprotected class drugs excluded from Medicare Part D formularies can only be covered via appeals,^3^ we accessed the Centers for Medicare & Medicaid Services (CMS) Appeals Decisions Search to identify common reasons for coverage denials and appeals decisions. Data were analyzed using SAS Studio version 9.4 (SAS Institute) and Microsoft Excel (Microsoft Corp). This study did not constitute human participants research, and, per the Common Rule, institutional review board approval and informed consent were not required. This study followed the STROBE reporting guideline.

## Results

Among the 11 nonprotected class drugs studied, 10 (90.9%) were covered by at least 1 Medicare Part D plan after accelerated approval. Median (IQR) time from accelerated approval to initial coverage was 212.0 (189.0-253.8) days. In the year of initial formulary inclusion, drugs were covered by 7915 of 38 531 available plans (20.5%) (115 of 5249 [2.2%] for seladelpar to 1880 of 3416 [55.0%] for bedaquiline) (Figure). Coverage varied over time, and only 3 of 11 drugs (27.3%) were covered by more than 95.0% of available plans (eg, droxidopa, bedaquiline, and amikacin liposome inhalation suspension in the 2, 3, and 6 years after initial formulary inclusion, respectively).

**Figure.**
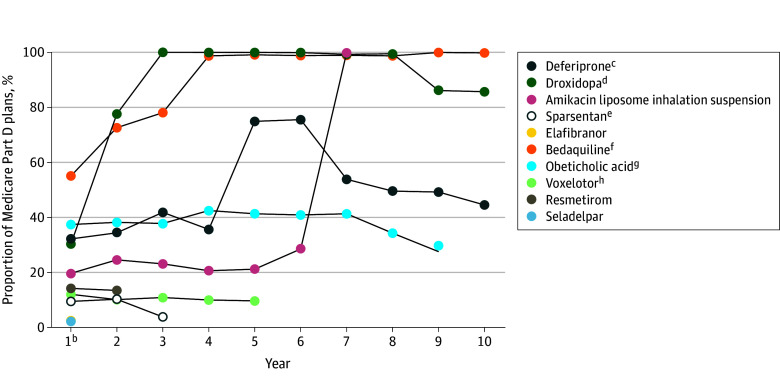
Trends in Medicare Part D Formulary Coverage of Nonprotected Class Drugs Granted Accelerated Approval^a^ ^a^Quarterly Medicare Prescription Drug Plan Formulary and Plan Information files were used to identify the proportion of plans that covered each nonprotected class drug granted accelerated approval. Migalastat was not covered by a Medicare Part D plan after accelerated approval. ^b^Year 1 is the year of initial Medicare Part D formulary inclusion. ^c^Deferiprone received additional accelerated approvals for iron overload due to thalassemia syndromes (September 9, 2015 [Year 4] and May 19, 2020 [Year 9]) and was converted to full approval on April 30, 2021 (Year 10). Generic competitors for deferiprone were approved in 2021 (Year 10). Coverage for deferiprone was 45% in 2022 (Year 11) and 2023 (Year 12) and declined to 42% and 33% in 2024 (Year 13) and 2025 (Year 14), respectively. ^d^Generic competitors for droxidopa were approved in 2021 (Year 7). Coverage of droxidopa was 86% in 2025 (Year 11). ^e^Sparsentan was converted to full approval on September 5, 2024 (Year 2). ^f^Bedaquiline received additional accelerated approvals for tuberculosis (August 9, 2019 [year 7] and May 27, 2020 [year 8]) and was converted to full approval on June 21, 2024 (year 12). Coverage for bedaquiline was 99% in 2023 (year 11) and 2024 (year 12) and 100% in 2025 (year 13). ^g^Competitors for obeticholic acid (specific to primary biliary cholangitis) were approved in 2024 (year 8). ^h^Voxelotor received an additional accelerated approval for sickle cell disease (December 17, 2021 [year 2]). The pharmaceutical manufacturer announced voluntarily withdrawal from the market in 2024 (year 5).

From 2020 to 2025, 443 Medicare Part D appeals decisions were documented for 10 drugs; 396 appeals decisions (89.4%) were unfavorable (6 of 10 [60.0%] for voxelotor to 4 of 4 [100.0%] for seladelpar and 2 of 2 [100%] for migalastat). The most common rationale for such decisions was off-label use (191 [48.2%]), followed by documentation not satisfying prior authorization requirements (93 [23.5%]) or formulary exception criteria (74 [18.7%]) (Table). Findings were similar for generic competitors of droxidopa and deferiprone: 247 unfavorable decisions (87.6%), of which 133 (53.8%) pertained to off-label use and 87 (35.6%) did not meet prior authorization requirements or formulary exception criteria.

**Table.** Medicare Part D Appeals Decisions and Common Rationale^a,b^

Drug	Approval date	Indication	No. of appeals	Appeals decisions	Common rationale of appeals decisions
Seladelpar	August 7, 2024	Primary biliary cholangitis	4 (Q4 2024)	4 Unfavorable	2 Off-label use/not medically accepted indication; 2 do not meet formulary exception
Resmetirom	March 14, 2024	Noncirrhotic nonalcoholic steatohepatitis	101 (Q2 2024 to Q1 2025)	95 Unfavorable	27 Off-label use/not medically accepted indication; 42 do not meet formulary exception; 25 do not support medical necessity for or do not meet prior authorization exception; 1 does not support step therapy exception
Sparsentan	February 17, 2023	Reduce proteinuria in primary immunoglobin A neuropathy	10 (Q3 2023 to Q1 2025)	8 Unfavorable^c^	2 Off-label use/not medically accepted indication; 4 do not meet formulary exception; 1 does not support medical necessity for or does not meet prior authorization exception; 1 does not support quantity limit exception
Voxelotor	November 25, 2019	Sickle cell disease	10 (Q1 2021 to Q2 2024)	6 Unfavorable	1 Off-label use/not medically accepted indication; 3 do not meet formulary exception; 2 ineligible for tiering exception
Amikacin liposome inhalation suspension	September 28, 2018	*Mycobacterium avium* complex lung disease	151 (Q1 2020 to Q4 2024)	139 Unfavorable^d^	93 Off-label use/not medically accepted indication; 17 do not meet formulary exception; 23 do not support medical necessity for or do not meet prior authorization exception; 1 does not meet coverage criteria; 1 ineligible for tiering exception; 2 cannot establish if drug is for medically accepted indication; 1 enrollee responsible for cost-sharing not applied at point of sale
Migalastat	August 10, 2018	Fabry disease	2 (Q2 2022 to Q2 2023)	2 Unfavorable	2 Do not meet formulary exception
Obeticholic acid	March 27, 2016	Primary biliary cholangitis	40 (Q1 2020 to Q3 2024)	32 Unfavorable	3 Off-label use/not medically accepted indication; 24 do not support medical necessity for or do not meet prior authorization exception; 3 ineligible for tiering exception; 1 does not support quantity limit exception; 1 paid correctly/no additional reimbursement from plan is required
Droxidopa^e^	February 18, 2014	Orthostatic dizziness or lightheadedness in symptomatic neurogenic orthostatic hypotension caused by primary autonomic failure	76 (Q1 2020 to Q4 2024)	70 Unfavorable	33 Off-label use/not medically accepted indication; 4 do not meet formulary exception; 13 do not support medical necessity for or do not meet prior authorization exception; 3 do not meet coverage criteria; 13 ineligible for tiering exception; 3 do not support quantity limits exception; 1 cannot establish if drug is for medically accepted indication
Bedaquiline	December 12, 2012	Pulmonary multi-drug resistant tuberculosis	36 (Q1 2020 to Q4 2024)	30 Unfavorable^f^	23 Off-label use/not medically accepted indication; 5 do not support medical necessity for or do not meet prior authorization exception; 1 does not meet coverage criteria; 1 does not meet step therapy exception
Deferiprone^g^	October 14, 2011	Transfusional iron overload due to thalassemia syndromes	13 (Q1 2020 to Q2 2024)	10 Unfavorable^h^	7 Off-label use/not medically accepted indication; 2 do not support medical necessity for or do not meet prior authorization exception; 1 ineligible for tiering exception

Abbreviation: Q, quarter.

^a^ Centers for Medicare & Medicaid Services Appeals Decision Search was used to identify unfavorable appeals decisions and associated common rationale from January 2020 to January 2025. In 2020 there were 23 738 appeals decisions (89.9% unfavorable), in 2021 there were 25 491 appeals decisions (95.0% unfavorable), in 2022 there were 34 771 appeals decisions (94.5% unfavorable), in 2023 there were 35 093 appeals decisions (93.5% unfavorable), in 2024 there were 44 812 appeals decisions (94.3% unfavorable), and in January 2025 there were 3910 appeals decisions (94.2% unfavorable). Appeals were reviewed and decided by Qualified Independent Contractors.

^b^ Elafibranor (Iqirvo) did not have appeals documented from June 2024 to January 2025.

^c^ Four unfavorable appeals decisions for sparsentan occurred after the drug was converted to traditional approval in September 2024 (1 off-label use/not medically accepted indication; 2 do not meet formulary exception; and 1 does not support quantity limit exception).

^d^ One unfavorable appeals decision for amikacin liposome inhalation suspension was missing an associated common rationale.

^e^ Generic competitors for droxidopa had 225 unfavorable appeals decisions, of which 114 were for off-label use, 84 did not meet coverage criteria (prior authorization requirements or formulary exclusion criteria), 13 cannot establish if drug is for medically accepted indication, 8 were ineligible for tiering exception, 5 did not support quantity limits exception, and 1 paid correctly/no additional reimbursement from plan is required.

^f^ Two unfavorable decisions for bedaquiline occurred after conversion to traditional approval in June 2024 (1 off-label use/not medically accepted indication; 1 does not meet step therapy exception).

^g^ Generic competitors for deferiprone had 22 unfavorable appeals decisions, of which 19 were for off-label use, 3 did not meet coverage criteria (formulary exception criteria), and 1 was ineligible for tiering exception.

^h^ Seven unfavorable decisions for deferiprone occurred after the drug was converted to traditional approval in April 2021 (6 off-label use/not medically accepted indication; 1 does not support medical necessity for or does not meet prior authorization exception).

## Discussion

Nearly all nonprotected class drugs were covered by Medicare Part D after accelerated approval. Consistent with prior research of novel therapeutics,^2,3^ we observed 50% or less of Part D plans initially and continually covered most nonprotected class drugs. Limited formulary inclusion could be due to the high price^3^ and uncertain clinical benefit^3^ of accelerated approval products, as well as in-class and generic competition.

Additionally, we observed that more than one-third of unfavorable appeals decisions pertained to documentation not satisfying specific coverage criteria for both branded and generic drugs. Given the administrative burden of prior authorization and formulary exclusion requirements,^6^ future research should understand the delays in access to and subsequent harms for beneficiaries requiring nonprotected class drugs that have received accelerated approval.

Limitations included potential variability in plan enrollment or benefit structure,^2^ underestimation of coverage denials (eg, decision not appealed), and limited generalizability to other novel therapeutics or non-Medicare prescription drug plans. Variability in formulary coverage may impede access to medications for Medicare Part D beneficiaries. CMS should enhance reviews of formularies to ensure adequate, timely coverage of newly approved nonprotected class drugs, especially those that address serious illnesses and unmet medical needs.

## Notes

Supplement 1.
eMethods.
**eTable. **Orally administered nonprotected class drugs granted accelerated approval from January 2011 to December 2024

Supplement 2.
Data Sharing Statement
