# Supplementary material for: Trends in Formulary Coverage of Nonprotected Class Drugs Granted FDA Accelerated Approval
Source: JAMA Netw Open. 2025 Oct 7;8(10):e2536089. doi: 10.1001/jamanetworkopen.2025.36089 (PMC12505173; doi:10.1001/jamanetworkopen.2025.36089)
Supplement: Supplement 1. — eMethods. eTable. Orally administered nonprotected class drugs granted accelerated approval from January 2011 to December 2024 [file jamanetwopen-e2536089-s001.pdf]

Supplemental Online Content

Jazowski SA, Dusetzina SB. Trends in formulary coverage of nonprotected class drugs granted FDA accelerated approval. *JAMA Netw Open*. 2025;8(10):e2536089. doi:10.1001/jamanetworkopen.2025.36089

eMethods.

eTable. Orally administered nonprotected class drugs granted accelerated approval from January 2011 to December 2024

This supplemental material has been provided by the authors to give readers additional information about their work.

© 2025 Jazowski SA et al. *JAMA Network Open*.

eMethods.

Data Sources

CDER Drug and Biologic Accelerated Approvals Based on a Surrogate Endpoint (as of December 31, 2024). https://www.fda.gov/drugs/nda-and-bla-approvals/accelerated-approval-program

Quarterly Prescription Drug Plan Formulary and Plan Information Files. https://data.cms.gov/provider-summary-by-type-of-service/medicare-part-d-prescribers/quarterly-prescription-drug-plan-formulary-pharmacy-network-and-pricing-information

Centers for Medicare & Medicaid Services Appeals Decisions Search. https://www.cms.gov/medicare/appeals-grievances/appeals-decision-search-part-c-d

Identification of Accelerated Approval Products and Formulary Coverage

Identification of Accelerated Approval Products

We used the CDER Drug and Biologic Accelerated Approvals Based on a Surrogate Endpoint Report (as of December 31, 2024)[1] to identify orally administered non-protected class drugs newly granted accelerated approval from January 2011 to December 2024 (n=16). We then reviewed the Drugs@FDA website and each product's label to identify additional indications. We excluded drugs that exclusively treated pediatric populations (nifurtimox and benznidazole) and if accelerated approval was for a supplemental indication (iptacopan, alpelisib, and budenoside). The final cohort of 11 drugs and associated accelerated approval indications can be found in the eTable in the Supplement.

Identification and Timing of Formulary Inclusion

We used RxNorm concept unique identifiers (RXCUI), which are available from the National Library of Medicine's RxNav website,[2] to identify Medicare Part D coverage in the quarterly Prescription Drug Plan Formulary and Plan Information files.[3] Since we used quarterly files, we assigned a date for when a product was first covered by a Medicare Part D plan. For example, if we first observed coverage in quarter 2 of 2020, then a date of April 1, 2020, was assigned. We then used this date to estimate the time from accelerated approval (date displayed in the CDER Drug and Biologic Accelerated Approvals Based on a Surrogate Endpoint Report and the eTable in the Supplement) to initial formulary inclusion.

References:

1. FDA. CDER Drug and Biologic Accelerated Approvals Based on a Surrogate Endpoint (as of December 31, 2024). Accessed July 28, 2025. https://www.fda.gov/drugs/nda-and-bla-approvals/accelerated-approval-program
2. National Library of Medicine. RxNav. Accessed July 28, 2025. https://mor.nlm.nih.gov/RxNav/
3. Data.CMS.gov. Quarterly prescription drugs plan formulary, pharmacy network, and pricing information. Accessed July 28, 2025. https://data.cms.gov/provider-summary-by-

© 2025 Jazowski SA et al. *JAMA Network Open*.

type-of-service/medicare-part-d-prescribers/quarterly-prescription-drug-plan-formulary-pharmacy-network-and-pricing-information

© 2025 Jazowski SA et al. *JAMA Network Open*.

eTable. Orally Administered Non-Protected Class Drugs Granted Accelerated Approval from January 2011 to December 2024

**Product[a,b]**	**Approval Date**	**Indication**	**Status**
Seladelpar	8/7/2024	Primary biliary cholangitis	Not yet converted
Elafibranor	6/10/2024	Primary biliary cholangitis	Not yet converted
Resmetirom	3/14/2024	Noncirrhotic nonalcoholic steatohepatitis	Not yet converted
Sparsentan	2/17/2023	Reduce proteinuria in primary immunoglobin A neuropathy	Converted (9/5/2024)
Voxelotor[c]	11/25/2019	Sickle cell disease	Withdrawn (9/25/2024)
Amikacin liposome inhalation suspension	9/28/2018	Mycobacterium avium complex lung disease	Not yet converted
Migalastat	8/10/2018	Fabry disease	Not yet converted
Obeticholic acid[d]	5/27/2016	Primary biliary cholangitis	Not yet converted
Droxidopa[e]	2/18/2014	Orthostatic dizziness, lightheadedness, or feeling about to black out in symptomatic neurogenic orthostatic hypotension caused by primary autonomic failure	Not yet converted
Bedaquiline[f]	12/12/2012	Pulmonary multi-drug resistant tuberculosis	Converted (6/21/2024)
Deferiprone[g]	10/14/2011	Transfusional iron overload due to thalassemia syndromes	Converted (4/30/2021)

[a] We reviewed the CDER Drug and Biologic Accelerated Approvals Based on a Surrogate Endpoint Report (as of December 31, 2024) to identify orally administered non-protected class drugs granted accelerated approval from January 2011 to December 2024. Of the 16 products that were newly granted accelerated approval, we excluded those that exclusively treated pediatric populations (nifurtimox and benznidazole) and received accelerated approval for a supplemental indication (iptacopan, alpelisib, and budesonide).

[b] We focused on non-protected class drugs because protected class drugs are required to be covered by Medicare prescription drug plans. We used the following World Health Organization Anatomical Therapeutic Chemical (ATC) codes to ensure selected products were not categorized as a protected class: L04 (immunosuppressants for prophylaxis of organ transplant rejection); N06A (antidepressants); N05A (antipsychotics), N03A (anticonvulsants); J05A (antiretrovirals); and L01 (antineoplastic agents).

[c] Voxelotor received multiple accelerated approvals for sickle cell disease (11/25/2019 and 12/17/2021). The pharmaceutical manufacturer announced voluntarily withdrawal from the market in 2024.

[d] Competitors for primary biliary cholangitis were approved in 2024.

[e] Generic competitors for droxidopa were first approved in 2021.

[f] Bedaquiline received multiple accelerated approvals for tuberculosis (12/12/2012, 8/9/2019, and 5/27/2020).

[g] Deferiprone received multiple accelerated approvals for iron overload due to thalassemia syndromes (10/14/2011, 9/9/2015, and 5/19/2020). Generic competitors for deferiprone were first approved in 2021.

© 2025 Jazowski SA et al. *JAMA Network Open*.
