# Supplementary material for: Trends in Formulary Coverage of Nonprotected Class Drugs Granted FDA Accelerated Approval
Source: JAMA Netw Open. 2025 Oct 7;8(10):e2536089. doi: 10.1001/jamanetworkopen.2025.36089 (PMC12505173; doi:10.1001/jamanetworkopen.2025.36089)
Supplement: Supplement 2. — Data Sharing Statement [file jamanetwopen-e2536089-s002.pdf]

Data Sharing Statement

Jazowski. Trends in Formulary Coverage of Nonprotected Class Drugs Granted FDA Accelerated Approval. *JAMA Netw Open*. Published October 07, 2025. doi:10.1001/jamanetworkopen.2025.36089

Data
Data available: No
